# D-Box Binding Protein (DBP) as a Circadian Output Regulator: Molecular Mechanisms, Tissue-Specific Functions, and Disease Relevance

**DOI:** 10.3390/ijms27125447

**Published:** 2026-06-16

**Authors:** Feng Liu, Jian-Xiang Cheng, Quan-Gang Wang, Zhong-Hong Wu, Yao Guo

**Affiliations:** State Key Laboratory of Animal Nutrition and Feeding, College of Animal Science and Technology, China Agricultural University, Beijing 100193, China; cauliufeng@163.com (F.L.); chengjx1997@163.com (J.-X.C.); pftzdwyzc@163.com (Q.-G.W.); wuzhh@cau.edu.cn (Z.-H.W.)

**Keywords:** D-box binding protein, circadian output, D-box, tissue-specific regulation

## Abstract

D-box binding protein (DBP) is a high-amplitude proline- and acidic amino acid-rich basic leucine zipper (PAR bZIP) transcription factor that functions as a key circadian output regulator downstream of the core molecular clock. Although DBP is widely recognized as a clock-controlled gene, its broader role in converting circadian timing into tissue-specific physiological programs remains incompletely integrated. In this review, we synthesize current evidence supporting DBP as a context-dependent D-box-centered regulatory node. We first summarize the upstream mechanisms that establish rhythmic *Dbp* expression, including CLOCK–BMAL1-dependent transcription, promoter-level amplification, signaling-dependent modulation, and post-translational control of DBP stability. We then discuss how DBP, together with related PAR bZIP activators and the opposing repressor E4 promoter-binding protein 4/nuclear factor interleukin 3 regulated (E4BP4/NFIL3), regulates D-box-mediated transcriptional output. Finally, we examine tissue-selective DBP functions in hepatic metabolism, pancreatic β-cell secretory competence, neural and behavioral regulation, reproductive neuroendocrine timing, and T helper 9 (Th9)-associated antitumor immunity. Across these systems, DBP does not act as a universal circadian effector; rather, its function depends on chromatin accessibility, cofactor availability, competing transcription factors, and local signaling context. We also highlight the current limits of human translational evidence and propose that DBP-centered signatures may be useful for interpreting circadian output failure in disease. Overall, DBP provides a mechanistically informative framework for understanding how circadian time is transformed into organ-specific physiological function and pathological vulnerability.

## 1. Introduction

Biological rhythms are endogenous timing programs that organize physiology across multiple temporal scales, including circadian (~24 h), ultradian (<24 h), and infradian (>24 h) rhythms [1,2]. Among these, circadian rhythms provide the principal temporal framework in mammals by aligning organism-level outputs, such as sleep–wake/rest–activity cycles, body temperature rhythms, and endocrine secretion, with cellular and molecular programs including metabolic regulation and rhythmic gene expression [3,4,5]. In this sense, circadian timing is not simply an accompanying feature of physiology, but a fundamental organizing principle that determines when physiological processes are optimally executed [6,7]. This temporal organization is increasingly challenged in modern environments by artificial light at night [8,9], shift work [10], sleep curtailment [11], and irregular eating patterns [12,13], all of which can promote circadian misalignment and increase cardiometabolic vulnerability [14,15].

**Figure 1:**
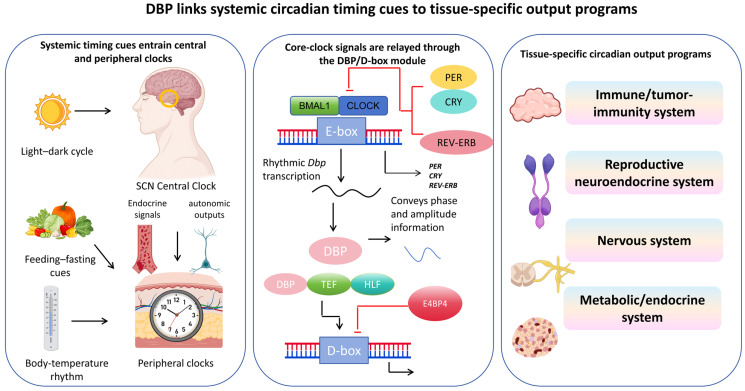
DBP as a relay linking systemic circadian timing cues to tissue-specific output programs. Environmental and behavioral cues synchronize the mammalian circadian hierarchy. Light primarily entrains the suprachiasmatic nucleus (SCN) central clock, whereas feeding-related cues strongly influence peripheral clocks, especially in metabolic tissues. The SCN further coordinates peripheral timing through systemic outputs, including neural, endocrine, behavioral, and physiological signals. At the cellular level, central and peripheral clocks are sustained by transcription–translation feedback loops, in which CLOCK:BMAL1-dependent E-box activation generates rhythmic clock-controlled transcription. DBP is positioned downstream of this core-clock machinery: rhythmic *Dbp* transcription leads to DBP accumulation, and DBP, together with TEF and HLF, promotes D-box-mediated transcription, whereas E4BP4/NFIL3 provides an opposing repressive input. Through this activation–repression module, DBP helps transmit circadian phase and amplitude information to tissue-selective output programs in immune/tumor-immunity, reproductive neuroendocrine, nervous, and metabolic/endocrine systems.

In mammals, circadian organization is structured as a hierarchical yet distributed timing system. The suprachiasmatic nucleus (SCN) acts as the central pacemaker and is primarily synchronized by retinal photic input, whereas self-sustained peripheral clocks are present in most organs and cell types [16,17]. Importantly, the SCN does not coordinate peripheral clocks through a single linear pathway. Instead, it distributes temporal information through multiple systemic routes, including direct and indirect neural outputs, autonomic nervous system activity, endocrine and humoral signals, daily body-temperature rhythms, and the behavioral organization of sleep–wake and feeding–fasting cycles [17,18,19]. Among endocrine signals, glucocorticoid rhythms are particularly important because they can act as systemic timing cues for peripheral oscillators, while SCN-driven autonomic regulation contributes to rhythmic adrenal sensitivity and hormone release [17,19,20]. Body-temperature cycles also provide a broadly acting timing signal: shallow physiological temperature rhythms can synchronize peripheral cellular clocks, whereas the SCN network itself is relatively resistant to temperature resetting, allowing the central pacemaker to impose temperature-based timing cues on peripheral tissues [17,21,22]. In parallel with these SCN-dependent outputs, peripheral clocks are also sensitive to local and behavioral signals. Feeding–fasting cycles are among the strongest non-photic zeitgebers for metabolic tissues; restricted feeding can shift liver and other peripheral clocks while the SCN remains largely locked to the light–dark cycle [15,23,24]. Feeding-related cues may include nutrient availability, glucose and fatty acids, cellular redox state, and postprandial hormonal signals such as insulin and IGF-1, which can reset cellular clocks by promoting PERIOD protein synthesis [23,24,25]. At the cellular level, these systemic and local timing signals converge on transcription–translation feedback loops (TTFLs), in which CLOCK and brain and muscle ARNT-like 1 (BMAL1) activate the transcription of *Period (Per)* and *Cryptochrome (Cry)* genes through E-box elements, and the resulting PER/CRY complexes subsequently repress CLOCK–BMAL1 activity to sustain an approximately 24 h oscillation [5,17,26]. Auxiliary interlocked loops, including those mediated by retinoic acid receptor-related orphan receptors (RORs) and REV-ERBs through ROR response elements (ROREs), further stabilize circadian phase and amplitude [5,17,27]. As summarized in Figure 1, circadian physiology therefore depends not only on the existence of central and peripheral oscillators, but also on the routing of temporal information through neural, endocrine, thermal, behavioral, metabolic, and transcriptional output pathways.

Although circadian biology is often introduced through the core transcription–translation feedback loop (TTFL) [28,29], physiological specificity ultimately depends on how core clock oscillations are transmitted to downstream transcriptional programs [5,30]. In addition to E-box- and ROR response element (RORE)-mediated regulation, other cis-regulatory modules [31], including cAMP response elements (CREs) [32], peroxisome proliferator response elements (PPREs), and D-box elements, provide interfaces through which circadian timing can be integrated with signaling pathways and metabolic state [33,34]. Among these, the D-box represents a particularly important output node [35]. D-box-dependent transcription is shaped by the opposing actions of PAR bZIP activators, especially DBP, together with TEF and HLF, and the repressor E4BP4/NFIL3 [36]. Through this module, the phase and amplitude of rhythmic downstream genes can be tuned in a context-dependent manner.

Within this framework, DBP should be viewed not merely as a peripheral clock-controlled gene, but as a circadian output regulator that links the core molecular oscillator to tissue-relevant transcriptional programs [35,37,38] (Figure 1). Its regulatory position is defined by two connected features: first, *Dbp* is rhythmically induced downstream of CLOCK:BMAL1-dependent transcriptional activity [35,39,40]; second, accumulated DBP acts through its bZIP domain to bind D-box cis-elements and regulate downstream targets, often in functional opposition to the D-box repressor E4BP4/NFIL3 [37,41,42]. Through this regulatory module, DBP can transmit circadian phase and amplitude information from the core clock to selected gene networks involved in metabolism, endocrine regulation, immune differentiation, reproductive neuroendocrine timing, and neural function [37,43,44,45,46]. Importantly, however, DBP-dependent output should not be interpreted as uniform across tissues. Available evidence suggests that its functional specificity is shaped by local chromatin accessibility, enhancer–promoter architecture, cofactor availability, signaling context, and competition or cooperation with other transcription factors [47,48,49].

Therefore, the goal of this review is to synthesize DBP biology as a context-dependent circadian output layer rather than as a single downstream clock-controlled gene. The novelty of this review lies in integrating three levels of evidence that are often considered separately: the rhythmic induction of *Dbp* by the core clock, D-box-mediated transmission of circadian timing signals to downstream targets, and the tissue-selective deployment of DBP-centered output pathways in physiological and disease-related contexts [16,37,39,47]. This framework is relevant not only for understanding circadian transcriptional architecture, but also for interpreting practical and translational problems [5,16,17]. In animal production, DBP-centered output may provide a useful conceptual link between environmental timing cues, such as light schedules and feeding time, and tissue-level changes in metabolism, endocrine function, reproduction, immunity, and stress-related physiological adaptation [50,51]. In clinical contexts, altered DBP/D-box-centered output may help explain how circadian misalignment is translated into metabolic dysfunction, β-cell failure, reproductive timing defects, neurobehavioral vulnerability, inflammation, and tumor-immunity-related phenotypes [52,53,54,55]. By integrating molecular studies with tissue-specific examples, this review aims to clarify how temporal information generated by the core clock is converted into organ-specific physiological programs and pathological vulnerability.

## 2. Methodological Considerations for Studying Circadian Rhythms and DBP-Centered Output

This review was designed as a narrative and mechanistic synthesis focused on DBP as a circadian output regulator. Relevant literature was identified through searches of PubMed, Web of Science, and Google Scholar using terms related to “D-box binding protein”, “DBP”, “PAR bZIP”, “D-box”, “E4BP4”, “NFIL3”, “CLOCK”, “BMAL1”, “circadian output”, “rhythmic transcription”, “phase-resolved sampling”, “tissue-specific circadian regulation”, “metabolism”, “pancreatic islet”, “reproductive neuroendocrinology”, “immune differentiation”, and “disease”. Methodologically, circadian studies require more than comparing two time points. A robust rhythm study should capture temporal structure, including period, phase, amplitude, and baseline level [56]. This usually requires phase-resolved sampling across at least one full circadian cycle, sufficient biological replication, and careful distinction between Zeitgeber time and circadian time when light–dark and constant-condition designs are used [56]. For molecular or genome-scale rhythm studies, sampling interval, total duration, replicate number, and the expected fraction of rhythmic signals strongly influence statistical power and biological interpretation. Time-series phenotyping may include behavioral activity, feeding rhythm, body temperature, hormone secretion, glucose and lipid metabolism, and tissue-specific molecular readouts. Real-time reporter systems, such as PER2::LUC or promoter–luciferase assays, provide complementary strategies for monitoring clock dynamics in cultured cells, tissue explants, and selected in vivo settings [57].

Rhythm detection also depends on appropriate statistical methods. Cosinor-based rhythmometry is useful when the expected waveform is approximately sinusoidal and provides interpretable parameters such as mesor, amplitude, and acrophase [58,59]. JTK_CYCLE is widely used for nonparametric detection of rhythmic components in large-scale datasets, whereas RAIN can detect more asymmetric waveforms [60]. MetaCycle integrates ARSER, JTK_CYCLE, and Lomb–Scargle methods and is useful for evaluating periodicity in large-scale time-series data [61]. However, rhythmic expression alone is not sufficient to establish DBP as a functional circadian output regulator [41]. For DBP-centered studies, stronger mechanistic evidence requires connecting time-resolved *Dbp* mRNA and DBP accumulation with D-box binding, transcriptional regulation, perturbation-based causality, and tissue-level function [35]. Useful approaches include phase-resolved quantitative PCR (qPCR) or RNA sequencing (RNA-seq), DBP profiling, D-box promoter-reporter assays with intact and mutated binding elements, chromatin immunoprecipitation qPCR/sequencing (ChIP-qPCR/ChIP-seq) for DBP, TEF, HLF, E4BP4/NFIL3, CLOCK, and BMAL1 occupancy, chromatin-accessibility assays such as assay for transposase-accessible chromatin using sequencing (ATAC-seq), and perturbation experiments targeting *Dbp*, *E4bp4*/*Nfil3*, related PAR bZIP factors, or TRAF7-mediated DBP turnover [39,41]. In this review, DBP-related evidence is therefore interpreted according to a stepwise framework: rhythmic expression, molecular binding and transcriptional activity, causal perturbation, and tissue-specific physiological relevance.

## 3. Molecular Regulatory Network of DBP: From Rhythmic Induction to Downstream Output

D-box binding protein (DBP; originally identified as albumin D-site-binding protein) belongs to the PAR bZIP (proline- and acidic amino acid-rich basic leucine zipper) family of transcription factors [62,63], and is best viewed as a circadian output regulator rather than as a constitutive component of the mammalian transcription–translation feedback loop (TTFL) itself [64]. This distinction is important. Much of circadian biology has historically been framed around the core oscillator, yet the physiological consequences of circadian timekeeping depend on how rhythmic information is conveyed to downstream genes that differ across tissues and functional systems [5]. DBP lies at precisely this interface. Its conserved bZIP domain provides the structural basis for this output function: the basic region mediates sequence-specific DNA recognition, whereas the leucine zipper supports dimerization, enabling DBP to recognize D-box cis-elements in the regulatory regions of target genes [37,65,66]. Through this mechanism, DBP does not generate the core oscillation, but instead interprets and redistributes oscillatory information into downstream transcriptional programs.

**Figure 2:**
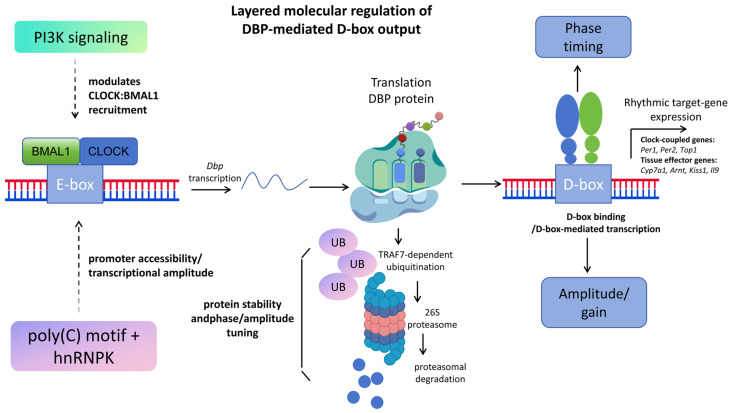
Layered molecular regulation of DBP-mediated D-box transcriptional output. This schematic summarizes the molecular regulatory pathway through which DBP converts core-clock timing into D-box-dependent transcriptional output. CLOCK:BMAL1 binding to E-box elements drives rhythmic *Dbp* transcription, while PI3K signaling modulates CLOCK:BMAL1 recruitment and the proximal poly(C) motif with hnRNP K supports promoter accessibility and transcriptional amplitude. After translation, DBP accumulation is further shaped by TRAF7-dependent ubiquitination and proteasomal degradation, indicating that DBP output is regulated at both transcriptional and post-translational levels. Accumulated DBP binds D-box elements, together with related PAR bZIP factors such as TEF and HLF, to promote rhythmic target-gene expression, whereas E4BP4/NFIL3 provides an opposing repressive input. This D-box-mediated regulatory layer influences both phase timing and amplitude/gain of downstream transcriptional rhythms, including clock-coupled genes such as *Per1*, *Per2*, and *Top1*, as well as tissue effector genes such as *Cyp7a1*, *Arnt*, *Kiss1*, and *Il9*.

Seen in this way, the regulatory logic of DBP can be divided into two connected processes: first, *Dbp* itself must be rhythmically induced by upstream clock machinery; second, once DBP accumulates, it must be deployed in a way that confers temporal structure on downstream genes. In the following sections, we therefore distinguish upstream mechanisms that establish rhythmic *Dbp* transcription, signaling and promoter-level mechanisms that tune its amplitude or phase, post-translational mechanisms that shape DBP accumulation, and downstream D-box mechanisms that transmit timing information to target genes. Figure 2 summarizes this logic as a stepwise framework extending from core-clock induction, through promoter- and protein-level tuning, to D-box-mediated output. This view is also consistent with classical work showing that circadian DBP transcription requires CLOCK-dependent regulation at the *Dbp* locus [35]. Rather than being a peripheral add-on to the clock, DBP should therefore be regarded as a major relay through which the phase and amplitude information generated by the core oscillator are transmitted to selective transcriptional outputs.

### 3.1. Upstream Rhythmic Induction of DBP

DBP is positioned immediately downstream of the core molecular oscillator. It is not a constitutive component of the mammalian TTFL in the same sense as BMAL1, CLOCK, PER, or CRY, but *Dbp* transcription is directly driven by core-clock activity. Classical work showed that CLOCK is required for circadian *Dbp* expression [35], and subsequent studies demonstrated rhythmic CLOCK:BMAL1 occupancy at multiple E-box motifs within the mouse *Dbp* locus, together with marked daily chromatin transitions [39,67]. These findings support the view that *Dbp* is a prototypical clock-controlled output gene rather than an autonomous core-clock gene. Later single-locus analyses further showed that BMAL1:CLOCK binding at *Dbp* is highly dynamic, strictly CLOCK dependent, and functionally tied to ongoing transcriptional activity [40,68,69].

This dynamic behavior is mechanistically important. Proteasome inhibition prolongs BMAL1:CLOCK residence at the *Dbp* locus but rapidly dampens *Dbp* transcription by reducing transcriptional burst frequency and burst size [40]. Thus, productive *Dbp* cycling depends on rapid turnover of the activator complex rather than on stable promoter occupancy alone. This point is important because it links CLOCK:BMAL1-dependent *Dbp* induction to the broader principle that rhythmic transcription requires dynamic transcription-factor exchange at target loci.

A second layer of control comes from local promoter context [70]. The proximal poly(C) motif in the *Dbp* promoter is now supported by direct functional evidence rather than by sequence inspection alone [70]. Deletion of this element weakens transcriptional activation, reduces chromatin accessibility around the promoter, and decreases RNA polymerase II recruitment, indicating that the poly(C) motif helps create a promoter state that can sustain strong rhythmic transcription. That function is reinforced by hnRNP K, which binds this proximal poly(C) region and promotes high-amplitude *Dbp* mRNA oscillation [71,72]. Taken together, these findings indicate that the high-amplitude rhythm of *Dbp* reflects cooperation between CLOCK:BMAL1-dependent activation and promoter-level amplification mechanisms, rather than being specified by core-clock input alone [49].

In addition to promoter architecture, intracellular signaling can modulate *Dbp* transcription. One relevant example is PI3K signaling. Morishita and colleagues reported that pharmacological inhibition or shRNA-mediated knockdown of PI3K blocked serum-shock-induced upregulation of *Dbp* mRNA, reduced *Dbp* promoter activity, decreased BMAL1/CLOCK recruitment to the E-box region of the *Dbp* promoter, and impaired BMAL1:CLOCK heterodimerization. These findings suggest that PI3K signaling acts as a modulatory input into CLOCK:BMAL1-dependent *Dbp* transcription, thereby linking cellular signaling state to a first-order clock-controlled output gene [73].

The phase and protein-level amplitude of DBP are also adjustable. In liver, CRY1 binds the *Dbp* promoter dynamically and delays BMAL1:CLOCK-driven transcription relative to other clock-controlled genes such as *Rev-erbα* [74]. Under changing photoperiods, this CRY1-dependent mechanism allows the phase of *Dbp* expression to shift while maintaining the alignment of peak DBP accumulation with the behavioral activity phase [74]. At the post-translational level, rhythmic DBP accumulation is shaped by regulated protein turnover. A recent study identified TRAF7 as a RING-type E3 ubiquitin ligase that promotes DBP ubiquitination and proteasome-dependent degradation; this pathway involves the E2 enzymes UBE2G1 and UBE2T, and altered TRAF7 abundance changes DBP levels and circadian period regulation [75]. Thus, rhythmic DBP accumulation is determined by a layered program involving E-box-driven transcription, promoter reinforcement, signaling modulation, phase tuning, and ubiquitin–proteasome-dependent protein stability.

### 3.2. DBP-Mediated Transmission of Circadian Timing to Downstream Genes

Once DBP accumulates, it transmits temporal information through the D-box regulatory layer [76]. This output function depends on the structural properties of the PAR bZIP family. The basic region of DBP mediates sequence-specific DNA recognition, whereas the leucine zipper supports dimerization, enabling DBP to bind D-box cis-elements and activate transcription [37,65,66]. However, D-box-dependent output is not determined by DBP alone. DBP functions together with the related activating PAR bZIP proteins TEF and HLF, whereas E4BP4/NFIL3 acts as an opposing repressor that recognizes related D-box sequences [41,77]. Earlier work showed that TEF and DBP display similar circadian and tissue-specific expression patterns but differ in target promoter preferences, indicating that PAR bZIP output is shaped by both shared DNA-binding logic and factor-specific promoter context [77]. The antagonism between DBP/PAR bZIP activators and E4BP4/NFIL3 has been demonstrated at both the expression and DNA-binding/transcriptional levels. In liver and SCN, E4BP4 and DBP cycle in near antiphase, and E4BP4 competes with PAR bZIP proteins for common binding sites while repressing transcription where DBP, TEF, and HLF activate it [41]. Therefore, the functional output of a D-box element is not determined simply by the presence of DBP. Instead, it depends on the relative abundance and phase of activating and repressing D-box factors, local chromatin accessibility, promoter or enhancer architecture, and the availability of tissue-specific cofactors. In cell-based rhythm assays, altering DBP or E4BP4 abundance changes the period of *Per1*- and *Per2*-promoter-driven oscillations, supporting the view that D-box regulation can shape rhythmic transcriptional timing rather than functioning only as a passive downstream output branch [37,78].

That shaping function is not limited to a few classical examples. Genome-wide analyses further support this interpretation. In mouse liver, ChIP-seq identified 1490 genomic regions commonly recognized by DBP and E4BP4, and transcriptomic analysis in *E4bp4*-deficient liver showed that E4BP4-mediated repression contributes substantially to circadian gene-expression rhythms [33,37]. These data move the D-box module beyond isolated promoter-level examples and support its role as a bona fide circadian output-regulatory layer. The downstream reach of this layer extends beyond canonical clock genes. For example, the mouse *Topoisomerase I* promoter contains two putative E-boxes and one DBP/E4BP4-binding D-box, and luciferase analyses showed that its circadian expression is regulated by clock-associated transcriptional mechanisms [33,79]. More broadly, studies in PAR bZIP triple-knockout mice showed that DBP, TEF, and HLF jointly regulate genes involved in xenobiotic detoxification and drug metabolism, including cytochrome P450 enzymes, carboxylesterases, and the constitutive androstane receptor [43,80].This indicates that DBP-dependent output is not restricted to refining clock-gene waveforms, but also carries circadian timing information into physiologically consequential pathways [80,81]. Phase and amplitude should be considered as related but separable dimensions of DBP-dependent output [75]. DBP can influence the phase of downstream transcription by providing a temporally restricted activating signal at D-box-containing regulatory regions, particularly when its rhythm is offset from that of E4BP4/NFIL3. However, DBP may also shape the amplitude, or transcriptional gain, of downstream rhythms. This amplitude control can arise from the unusually high-amplitude rhythm of *Dbp* itself, promoter-level amplification mechanisms such as the proximal poly(C) motif and hnRNP K, local chromatin accessibility, and the relative abundance of activating PAR bZIP factors versus the repressive E4BP4/NFIL3 arm. Thus, DBP should not be viewed only as a phase relay; in specific regulatory contexts, it may also determine how strongly a rhythmic transcriptional program is expressed [37,39,71].

The D-box layer also appears to sit at an interface where output and resetting can meet [37]. Activation of TGF-β/activin signaling resets cellular clocks through rapid induction of *Dec1* transcripts without requiring acute *Per* induction, showing that non-photic signals can enter the clockwork by routes other than the canonical light–*Per* pathway [82,83]. In parallel, systematic analysis of D-box function showed that acute induction of E4BP4 can evoke phase-dependent phase shifts and that D-box-mediated transcription contributes to both input and output in the circadian system [37,78]. It is therefore more accurate to view DBP/E4BP4-linked D-box regulation not as a one-way terminal branch, but as a selective regulatory layer where core timing, tissue context, and environmental or disease-associated signals can converge.

## 4. DBP as a Tissue-Selective Effector of Circadian Physiology and Disease

**Figure 3:**
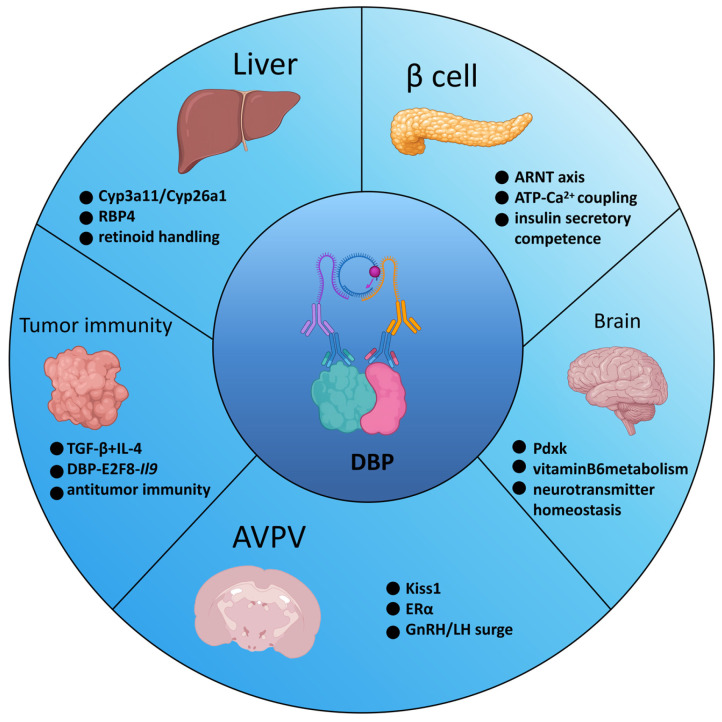
Tissue-selective deployment of DBP-centered circadian output in physiology and disease. This schematic summarizes how a shared DBP-centered D-box module can be deployed in a tissue-selective manner. In the central module, DBP, TEF, and HLF act as activating PAR bZIP factors, whereas E4BP4/NFIL3 provides an opposing repressive input; the functional consequence of this balance is shaped by chromatin accessibility, cofactors, and local signaling context. The outer sectors highlight representative tissue-specific circuits. In the liver, DBP-related output is linked to *Cyp3a11*/*Cyp26a1*, RBP4, and retinoid handling. In pancreatic β cells, the DBP/E4BP4–ARNT axis connects circadian output to ATP–Ca^2+^ coupling and insulin secretory competence. In the brain, the broader PAR bZIP layer is associated with *Pdxk*, vitamin B6 metabolism, and neurotransmitter homeostasis. In the AVPV, DBP contributes to *Kiss1*-linked reproductive neuroendocrine timing and the GnRH/LH surge. In Th9 cells, DBP opposes E2F8 to promote *Il9* transcription and antitumor immunity. Together, these examples illustrate that DBP-dependent output is not uniform, but is recruited into distinct tissue circuits with different physiological and disease-related consequences.

A recurring difficulty in interpreting DBP is that its biological importance is not best judged by asking whether it sustains the core oscillator, but by asking where it becomes functionally consequential downstream of the clock [84]. In this sense, DBP is more usefully viewed as a tissue-selective circadian effector than as a general regulator of rhythmicity [43,44,45,46,85]. As summarized in Figure 3, the impact of DBP is not distributed uniformly across the organism. Rather, it emerges in specific physiological settings in which rhythmic transcription must be translated into locally meaningful outputs, including metabolism, endocrine coordination, neural homeostasis, reproductive timing, and immune regulation [43].

The best-supported examples already suggest that this selectivity is biologically consequential. In liver, DBP has long been linked to circadian control of metabolic genes, including cholesterol 7α-hydroxylase, and more broadly to detoxification programs regulated by the PAR bZIP family [80,81]. In pancreatic islets and β cells, local clock function is required for rhythmic insulin secretion and normal glucose-stimulated insulin secretion, while the DBP/E4BP4 output axis regulates *Arnt*/*Hif-1β* and is disrupted under diabetic or chronic endoplasmic reticulum stress conditions [86,87]. In brain, the loss of DBP together with TEF and HLF produces marked neurological consequences, including spontaneous and audiogenic seizures, in association with disturbed vitamin B6 and neurotransmitter metabolism [88]. In reproductive neuroendocrine circuits, DBP can activate *Kiss1* transcription through a D-box-dependent mechanism in the AVPV, where circadian, estrogenic, and neuropeptidergic signals converge to gate the preovulatory GnRH/LH surge [45]. In immune biology, the picture is somewhat different. Strong evidence links E4BP4/NFIL3, the D-box antagonist of DBP, to TH17 differentiation and innate lymphoid cell development, whereas direct evidence for DBP itself is strongest in Th9 differentiation and antitumor immunity [46,89,90].

### 4.1. Mechanistic Basis of DBP Tissue Specificity

The tissue selectivity of DBP is unlikely to be explained by the presence or absence of DBP alone. Instead, available evidence supports a multilayered model in which DBP-dependent output is determined by the integration of rhythmic DBP abundance, local regulatory architecture, antagonistic D-box factors, tissue-specific signaling cues, and the identity of available target-gene networks [37,39,43,70,71]. First, *Dbp* expression itself can differ across tissues in phase, amplitude, and responsiveness to systemic cues, meaning that the temporal window of DBP availability is not identical in all organs [39]. Second, D-box motifs are functional only when embedded in a permissive chromatin and enhancer–promoter environment [37,70]. Thus, the same or similar D-box sequence may support transcriptional output in one tissue but remain inaccessible or functionally silent in another. Third, DBP acts within a competitive D-box module rather than as an isolated activator [37,41]. The relative phase and abundance of DBP, TEF, HLF, and the opposing repressor E4BP4/NFIL3 can determine whether a D-box-containing regulatory region is activated, repressed, or temporally reshaped [41].

Upstream RORE-dependent regulation may further contribute to this tissue-selective landscape. RORα and RORγ regulate clock-gene expression in an isotype- and tissue-selective manner, and loss of RORα/RORγ reduces the peak expression of several clock-related genes, including *Cry1*, *Bmal1*, *E4bp4*, *Rev-erbα*, and *Per2*, without necessarily shifting their phase [91,92,93]. This suggests that the ROR/REV-ERB–RORE layer can influence the amplitude and availability of upstream clock and D-box regulators in a tissue-dependent manner, rather than acting as a simple direct upstream switch for DBP alone [41,91]. This tissue-dependent RORE context is especially relevant to metabolic and immune organs, where ROR/REV-ERB-regulated clock outputs intersect with lipid/glucose metabolism, inflammatory differentiation, and organ-specific stress responses [91,94].

Finally, tissue-specific signaling and cofactor context determine which DBP-centered circuits become physiologically relevant. For example, TGF-β-associated signaling modifies hepatic DBP output in chronic kidney disease-related retinoid metabolism [85], chronic endoplasmic reticulum stress shifts the DBP/E4BP4 balance in β cells [95], estrogen/ERα cooperates with DBP in the AVPV *Kiss1* system [45], and TGF-β plus IL-4 recruits DBP into the Th9 *Il9* transcriptional program [46]. These examples indicate that tissue specificity arises not from a universal DBP target list, but from the intersection between circadian timing, local regulatory accessibility, competing transcription factors, and tissue-defined physiological programs. From this perspective, the critical question is not only where *Dbp* is rhythmic, but where DBP is recruited into an accessible and signal-competent regulatory circuit that controls functionally relevant genes. To make this conceptual framework explicit, Table 1 summarizes the major mechanistic determinants that may shape tissue-selective DBP output and links each determinant to representative examples discussed in the following sections.

### 4.2. Nervous System: DBP as a Stabilizer of Behavioral Output and Stress-Sensitive Neurobiological States

Early genetic studies in mice provided strong evidence that DBP contributes to circadian phenotypes in the nervous system, although not in the manner expected for an essential core clock component. *Dbp^−/−^* mice remain circadian rhythmic, but exhibit reduced locomotor activity and a shorter free-running period, indicating that DBP is not required for rhythm generation per se [84]. More prominent changes are observed in the quality and temporal organization of circadian output at the behavioral and EEG levels. In sleep recordings, *Dbp^−/−^* mice show roughly normal total sleep time, yet the day–night amplitude of sleep is reduced and sleep episodes are less consolidated under both light–dark and constant-dark conditions [97,98]. Quantitative EEG analyses further show that the normal sleep–wake-dependent variation in slow-wave activity is blunted, hippocampal theta peak frequency is shifted upward, and after sleep deprivation the rebound of REM sleep is attenuated even though the compensatory rebound of EEG delta power is largely preserved [97,99]. Taken together, these findings support the view that DBP primarily helps stabilize and shape the temporal structure of sleep–wake output rather than serving as a core pacemaker required for the generation of circadian rhythms [76].

A fuller picture emerges when DBP is considered within the broader PAR bZIP transcriptional layer in which it functions [100]. The strongest neural phenotypes become apparent when redundancy within this family is removed: mice lacking DBP together with TEF and HLF are highly susceptible to spontaneous and audiogenic seizures, and their brains contain reduced pyridoxal phosphate, serotonin, and dopamine, changes linked to dysregulation of *Pdxk*, a PAR bZIP target gene required for vitamin B6-dependent neurotransmitter metabolism [88,101]. These findings indicate that the PAR bZIP output layer that includes DBP contributes to neurochemical homeostasis, not merely to behavioral timing [88]. A second line of evidence comes from stress-reactive paradigms centered more directly on DBP itself. In a convergent functional genomics study, *Dbp* was prioritized as a candidate relevant to bipolar disorder and alcoholism, and *Dbp-null* mice displayed lower baseline locomotor activity, blunted responses to stimulants, reduced weight gain, activation by sleep deprivation that was prevented by valproate, and increased alcohol intake after stress exposure [102,103]. More recently, a chronic periodontitis model extended this argument into the neuroimmune domain: DBP was identified as a prominently upregulated hippocampal gene, was preferentially detected in microglia, and microglial DBP knockdown alleviated depression-like behavior, memory deficits, synapse loss, neurogenesis impairment, and inflammatory activation [104,105]. Taken together, current evidence does not support treating DBP as a disease-specific biomarker for any single neuropsychiatric condition; rather, it is more consistent with DBP being part of a circadian output layer that becomes particularly important when neural systems are challenged by stress, inflammation, or impaired neurochemical homeostasis [88,102,106].

### 4.3. Metabolic Systems: DBP as a Coordinator of Hepatic Retinoid Metabolism and Islet Output Competence

The liver provides some of the clearest evidence that DBP functions as a circadian output factor with direct metabolic consequences. In 5/6 nephrectomy models of chronic kidney disease, hepatic DBP is reduced together with *Cyp3a11* and *Cyp26a1*, two key DBP-linked enzymes in retinoid metabolism, and this shift is accompanied by altered retinoid metabolism, accumulation of circulating retinol, and worsening renal dysfunction [85]. The same study further showed that elevated TGF-β1 suppresses hepatic *Dbp* through a TCF7L2–ID2-associated pathway; anti–TGF-β1 treatment restored hepatic *Dbp*, *Cyp3a11*, and *Cyp26a1* expression and improved both retinoid metabolism and renal dysfunction, whereas hepatic *Dbp* restoration directly rescued hepatic *Cyp3a11*/*Cyp26a1* expression and reduced serum retinol accumulation [85]. Beyond retinoid handling, hepatic DBP also participates in hepatokine-mediated glucose regulation. RBP4 displays diurnal oscillation in both liver and plasma, and this rhythmicity is damped in liver-specific *Bmal1* knockout mice; BMAL1 regulates hepatic RBP4 expression through DBP. Knockdown of either hepatic *Dbp* or *Rbp4* increases insulin sensitivity in a time-of-day-dependent manner, whereas hepatic RBP4 overexpression reverses the insulin-sensitizing effect of liver-specific *Bmal1* deficiency [96]. Earlier promoter and transactivation studies had already shown that DBP contributes directly to circadian transcription of cholesterol 7α-hydroxylase (*Cyp7a1*), the rate-limiting enzyme in the conversion of cholesterol to bile acids [81], and later work identified *Cyp2a4* and *Cyp2a5* as additional hepatic DBP-regulated circadian targets [107]. At the broader PAR bZIP level, combined loss of DBP, TEF, and HLF disrupts multiple hepatic detoxification pathways, including genes encoding cytochrome P450 enzymes, carboxylesterases, and constitutive androstane receptor [43]. Nutrient timing also acts on this output layer: under parenteral nutrition, intermittent nocturnal feeding preserves the normal phase relationship of hepatic DBP and CYP7 expression, whereas intermittent diurnal feeding reverses both [108]. Collectively, these findings place DBP, and the broader PAR bZIP output layer in which it operates, at a metabolically important junction where clock timing, feeding schedule, cytokine signaling, retinoid turnover, bile-acid synthesis, hepatokine output, and xenobiotic metabolism converge.

In pancreatic islets, available evidence supports a relatively selective role for DBP in maintaining β-cell stimulus–secretion coupling and secretory readiness rather than acting as a universal regulator of all rhythmic genes. Circadian regulation is embedded in the islet secretory apparatus itself: pancreas-specific *Bmal1* deletion impairs glucose tolerance and glucose-stimulated insulin secretion, and isolated islets from these mice respond poorly not only to glucose but also to KCl, exendin-4, forskolin, and 8-Br-cAMP [86]. Complementing this, isolated mouse and human islets display intrinsic rhythmic insulin secretion synchronized with rhythmic expression of genes encoding secretory machinery and signaling factors, and adult β-cell clock ablation causes constitutively low insulin secretion and severe glucose intolerance [87]. Within this framework, DBP has been shown to activate *Arnt* (encoding HIF-1β) transcription in pancreatic β cells, whereas E4BP4 acts in the opposite direction at the same promoter [44]. This regulatory axis is functionally relevant because ARNT is markedly reduced in human type 2 diabetic islets, and ARNT loss impairs insulin secretion in both β-cell models and β-cell-specific knockout mice [109]. Consistent with a shift in the D-box layer away from DBP-dependent activation, diabetic islets from *Wfs1* mutant mice show reduced *Dbp*, increased *E4bp4*, and decreased *Arnt* [44]. Chronic endoplasmic reticulum stress sharpens this model further: ER stress decreases *Dbp*, increases *E4bp4*/*Nfil3*, and reduces DBP-dependent transcriptional activity [110]; in β-cell-specific E4BP4 transgenic mice, this imbalance is accompanied by marked glucose intolerance, severely impaired insulin secretion, elevated basal ATP/ADP ratios with loss of the normal circadian oscillation, failure of glucose to raise ATP/ADP or intracellular Ca^2+^ appropriately, and reduced expression of genes linked to insulin secretion, including *Ins1*, *Ins2*, *Slc2a2*, and *Rab37* [95]. Collectively, current evidence supports DBP as a selective circadian output factor that helps preserve β-cell secretory competence by linking clock timing to nutrient-responsive metabolism, Ca^2+^ signaling, and expression of genes required for insulin release.

### 4.4. Reproductive Neuroendocrine Timing: DBP as a Gatekeeper of the Preovulatory Permissive Window

A particularly clear example of tissue-selective DBP function is found in the female reproductive neuroendocrine axis, where the preovulatory LH surge depends on the convergence of estradiol positive feedback with an appropriate circadian phase. In rodents, AVPV kisspeptin neurons are a major integrative node in this process: AVPV *Kiss1* expression increases under estradiol positive feedback, many AVPV *Kiss1* neurons exhibit Fos activation at surge time, and central blockade of kisspeptin signaling suppresses or prevents the estrogen-induced/preovulatory GnRH/LH surge [111,112,113]. Within this framework, Xu and colleagues provided direct molecular evidence that DBP can activate mouse *Kiss1* transcription through a D-box-dependent mechanism and that this action is augmented by ERα/estrogen signaling. Importantly, they further showed that in the rat AVPV, *Dbp* mRNA displays a robust diurnal rhythm on proestrus but not on diestrus 1, and that some AVPV cells coexpress DBP and ERα [45]. Together, these findings support a model in which DBP contributes to coupling circadian phase information to estrogen-sensitive *Kiss1* transcription in the AVPV, rather than establishing DBP as the sole trigger of ovulation. Disruption of this D-box-linked timing mechanism would be expected to impair the temporal coordination between estradiol positive feedback, AVPV *Kiss1* activation, and GnRH/LH surge generation, thereby providing a plausible route through which circadian output failure could compromise ovulatory timing and reproductive competence.

The significance of DBP in this context may lie less in simple transcriptional activation per se than in its contribution to temporal gating. Across rodent models, additional studies collectively suggest that the preovulatory permissive window is assembled from multiple circadian checkpoints distributed across the AVPV–kisspeptin–GnRH axis [45]. The AVPV itself exhibits circadian oscillation of core clock genes, and in estradiol-treated females these rhythms are synchronized with rhythmic expression of *Kiss1* and *Avpr1a*, consistent with a local integrative role for this nucleus [111,114]. AVPV kisspeptin neurons also display rhythmic PER1 expression, and AVPV explants from PER2::LUC mice sustain endogenous circadian oscillations whose period is lengthened by estradiol, supporting local oscillatory properties within this circuit [115]. At the afferent level, SCN-derived vasopressinergic projections target preoptic kisspeptin neurons [116], and AVP can directly excite these neurons; importantly, this AVP responsiveness is permitted by estradiol [117]. At the efferent end, the GnRH system itself exhibits pronounced time-of-day-dependent sensitivity to kisspeptin stimulation, responding most robustly in the afternoon, when the preovulatory surge normally occurs [118]. Taken together, these findings support a restrained but mechanistically coherent conclusion: DBP does not define the entire preovulatory gate on its own, but forms part of the transcriptional machinery that helps establish the permissive state of the AVPV kisspeptin node, thereby contributing to the narrow temporal window in which estradiol positive feedback can be converted into surge-like neuroendocrine output.

### 4.5. Immune Differentiation and Tumor Immunity: Redeployment of DBP in the DBP–E2F8–IL-9 Axis

A well-defined example of DBP function outside the more classical metabolic and neuroendocrine circadian contexts is found in T helper 9 (Th9) differentiation. Th9 cells were originally identified as IL-9-producing CD4^+^ T cells induced under the combined influence of TGF-β and IL-4 [119,120]. Within this lineage, Park and colleagues identified DBP and E2F8 as opposing regulators of *Il9* transcription, with DBP acting as a transcriptional activator and E2F8 as a repressor [46]. Mechanistically, TGF-β plus IL-4 induced p38-dependent phosphorylation of the linker Ser213 residue of Smad3, and this pSmad3L-Ser213 state was necessary and sufficient for *Il9* transcriptional activation in their experimental system [46]. Under the same conditions, DBP expression increased, E2F8 expression decreased, and direct binding at the *Il9* promoter shifted toward increased DBP and reduced E2F8 occupancy [46]. Because E2F8 is an atypical E2F family repressor originally characterized in the context of cell-cycle-regulated transcription rather than circadian regulation [46,121], the DBP–E2F8 opposition places DBP within a broader differentiation-associated transcriptional network rather than within an isolated clock-output branch [121].

The functional significance of this pathway extends beyond in vitro differentiation. In the same study, Th9 cells in which *Dbp* was knocked down showed reduced antitumor activity, whereas knockdown of *E2f8* had the opposite effect in mouse melanoma and fibrosarcoma models, linking the DBP–E2F8–IL-9 axis to Th9-mediated tumor control in vivo [46]. This interpretation is reinforced by earlier studies showing that Th9 cells and IL-9 can mediate antitumor immunity in vivo. In particular, tumor-specific Th9 cells promoted CCL20/CCR6-dependent recruitment of dendritic cells into tumor tissues, followed by tumor-antigen delivery to draining lymph nodes and downstream CD8^+^ T cell priming [122]. Complementing this, exogenous IL-9 inhibited tumor growth, and adoptive transfer of tumor-antigen-specific Th9 cells suppressed melanoma growth in an IL-9-dependent manner, supporting a causal contribution of the Th9/IL-9 module to antitumor immunity [123]. What makes this DBP-centered axis especially informative is not that it places DBP within a putative core immune clock, but that it shows how a factor classically discussed as a circadian output protein can be redeployed in a highly specific immune differentiation program with measurable consequences in the tumor microenvironment. At present, the most defensible interpretation is that DBP contributes to tumor immunity here through its role in sustaining Th9 differentiation and IL-9 production, rather than justifying its classification as a general immune regulator across lymphocyte states.

### 4.6. Disease Relevance and Human Translational Evidence

Although the strongest causal evidence for DBP-centered output still comes from animal and cellular models, the disease relevance of this regulatory layer can be considered across several levels [46,85]. In metabolic disease, hepatic DBP has been linked to retinoid handling in chronic kidney disease models, while the β-cell DBP/E4BP4–ARNT axis connects circadian output to insulin secretory competence and endoplasmic reticulum stress-associated β-cell dysfunction [44,95]. In neurobiological disease contexts, loss of the broader PAR bZIP layer produces seizure susceptibility, and altered DBP signaling has been implicated in stress- and inflammation-related behavioral phenotypes [88,102,104]. In cancer and immunity, direct mechanistic evidence is strongest for the DBP–E2F8–IL-9 axis in Th9 differentiation and antitumor immunity [46,123], whereas broader human cancer studies more generally support disruption of circadian clock-gene expression as a feature of tumor progression rather than establishing DBP as a tumor-specific driver [124,125,126]. Cardiovascular relevance should also be interpreted cautiously: circadian disruption is clearly linked to cardiovascular physiology and disease risk, but direct DBP-specific cardiovascular mechanisms remain less well defined [127,128,129].

Human translational evidence for DBP itself remains limited and should therefore be framed conservatively. The human DBP gene encodes a D-box binding PAR bZIP transcription factor, and public resources document its expression across human tissues and cancer datasets, but these data are primarily associative rather than causal. Direct human genetic evidence should also be interpreted cautiously. A targeted search of the genome-wide association study (GWAS) Catalog did not identify a well-established DBP-specific disease mechanism comparable to the mechanistic animal and cellular evidence summarized above; interpretation is further complicated by the abbreviation “DBP,” which is also widely used for diastolic blood pressure and vitamin D-binding protein. Human circadian-disruption studies nevertheless provide relevant context: insufficient sleep reduces the amplitude of circadian rhythms in the human blood transcriptome, and mistimed sleep reduces the proportion of rhythmic blood transcripts from 6.4% at baseline to 1.0% under forced desynchrony, indicating that circadian output failure can be detected at the transcriptomic level [130,131]. Future translational work should therefore test whether DBP-centered signatures can serve as markers of circadian output integrity in human tissues, rather than assuming that DBP is already established as a standalone disease biomarker. To make these disease-related and translational distinctions explicit, Table 2 summarizes representative DBP- or PAR bZIP-centered disease contexts, the current level of evidence, and the degree to which each association can be interpreted as mechanistic or translational.

## 5. Integrative Summary: DBP as a Context-Dependent Circadian Output Node

Taken together, the evidence reviewed above supports a unified view of DBP as a context-dependent circadian output node rather than as a uniformly acting downstream clock component. At the molecular level, DBP is positioned downstream of the core transcription–translation feedback loop, where rhythmic CLOCK:BMAL1-dependent activation of the *Dbp* locus, promoter-level amplification, chromatin accessibility, signaling-dependent modulation, and post-translational control together determine the timing and amplitude of DBP availability. Once DBP accumulates, its output is transmitted mainly through D-box-containing regulatory elements, where DBP and related PAR bZIP activators, including TEF and HLF, act in opposition to the repressive factor E4BP4/NFIL3. Thus, DBP-dependent transcription is not dictated simply by the presence of a D-box motif or by rhythmic *Dbp* expression alone, but emerges from the interaction between rhythmic activator abundance, competing repressors, local enhancer–promoter architecture, chromatin permissiveness, and tissue-specific cofactors [39,41,70,75].

This context-dependent framework helps explain why DBP has different biological consequences across tissues. In the liver, DBP-centered output is linked to metabolic and detoxification programs [43,81]; in pancreatic β cells, it contributes to secretory competence through the DBP/E4BP4–ARNT axis [44,95]; in the nervous system, it helps shape behavioral, electrophysiological, and neurochemical outputs [88,97,104]; in the reproductive neuroendocrine system, it participates in AVPV *Kiss1*-linked temporal gating [45,132]; and in Th9 differentiation, it promotes *Il9*-associated antitumor immunity [46,122,123]. These examples indicate that DBP becomes functionally consequential when rhythmic transcription factor availability coincides with accessible regulatory elements, appropriate cofactors, and tissue-defined target-gene networks. From a disease and translational perspective, current evidence remains strongest in animal and cellular models, and direct human evidence is still limited. Therefore, DBP should not yet be viewed as a standalone disease biomarker or universal therapeutic target, but rather as a mechanistic indicator of how circadian timing information is translated into tissue-specific physiological function [130,131,133].

## 6. Future Perspectives: Why DBP Matters as a Circadian Output Node

Recent work has shifted the central question in DBP biology. The issue is no longer whether *Dbp* is rhythmic, but how DBP acquires regulatory specificity once it is positioned downstream of the core oscillator. Existing studies already indicate that this specificity is built in layers rather than inherited passively from the clock. At the *Dbp* locus itself, rhythmic BMAL1:CLOCK occupancy is coupled to marked chromatin transitions [39], and productive transcription depends on highly dynamic factor turnover [40]. The unusually high amplitude of *Dbp* expression is further reinforced by local promoter features such as the proximal poly(C) motif and hnRNP K [70,71], whereas downstream D-box output is shaped by competitive repression from E4BP4 on a genome-wide scale. More recently, post-translational control has also entered the picture, with TRAF7-dependent ubiquitination shown to regulate DBP stability and circadian period [75]. Taken together, these findings suggest that future work should move beyond cataloguing rhythmic expression and instead define how chromatin context, cofactor availability, antagonistic repressors, and protein turnover determine where DBP becomes functionally effective and where it does not.

A second priority is to resolve causality in time, not simply association at endpoints. The most informative disease-linked studies so far have placed DBP in concrete tissue circuits: hepatic retinoid metabolism and hepatokine signaling in chronic kidney disease [85,96], β-cell failure under chronic endoplasmic reticulum stress [44,95], *Kiss1* regulation in the AVPV [45], and Th9 differentiation in tumor immunity [46]. Yet even in these better-defined examples, it often remains unclear whether altered DBP activity is an initiating event, a permissive condition, or a secondary consequence of broader network disruption. That ambiguity is partly methodological. Many influential studies still rely on selected phases, endpoint phenotypes, or bulk tissue measurements. Inducible tissue-specific perturbation, phase-resolved sampling, and the growing set of single-cell circadian transcriptomic approaches should make it increasingly possible to identify when DBP-dependent output first diverges from an otherwise still-rhythmic system and in which cell populations that divergence begins [133,134,135].

A third task is to understand DBP as a point of environmental and systemic integration rather than as a fixed transcriptional relay [37]. The studies discussed in earlier sections already show that DBP-centered output can be reshaped by feeding time [136,137], TGF-β-driven inflammatory signaling [85], chronic ER stress [95], estradiol-dependent reproductive state [45], and lineage-defining immune cues such as TGF-β plus IL-4 [46]. In that respect, DBP sits at a useful intersection between clock time and physiological context. This is particularly relevant for circadian misalignment states, where timing cues may still be present but are no longer translated into the appropriate local output [127,138]. Future work should therefore ask how environmental schedules and disease-associated signals are integrated into DBP-dependent transcription across organs, and whether different forms of circadian disruption converge on common DBP-centered failure modes or instead produce tissue-specific output defects [139].

From a translational standpoint, the near-term value of DBP is more likely to lie in interpretation than in immediate therapeutic targeting. The evidence accumulated so far argues against treating DBP as a universal effector that can be manipulated in the same way across tissues. Its actions are shaped by local promoter architecture, competing repressors such as E4BP4 and E2F8, and the physiological programs into which it is recruited. For that reason, DBP-centered signatures may prove most useful as indicators of circadian output failure, helping distinguish disorders driven primarily by defective output deployment from those caused by more proximal clock disruption. Direct intervention at the DBP layer remains an interesting possibility, particularly where antagonistic axes such as DBP–E4BP4 or DBP–E2F8 are already mechanistically defined, but any such strategy will need to be developed with careful attention to tissue specificity, phase dependence, and off-target consequences. In that sense, DBP matters because it offers a tractable way to study how circadian time is converted into organ-level physiology and, under pathological conditions, into organ-specific failure.

## 7. Conclusions

DBP is best understood as a circadian output regulator that links core-clock timing to tissue-selective transcriptional programs rather than as a universal component of the core oscillator. Its function is established through layered regulation, including rhythmic CLOCK–BMAL1-dependent *Dbp* induction, promoter and chromatin context, D-box binding, competition with E4BP4/NFIL3, tissue-specific signaling inputs, and post-translational control of DBP stability. These mechanisms explain why DBP-dependent output differs across hepatic metabolism, pancreatic β-cell function, neural homeostasis, reproductive neuroendocrine timing, and Th9-mediated immunity. Future studies should move beyond documenting rhythmic *Dbp* expression and instead test causal DBP-dependent output through phase-resolved sampling, tissue-specific perturbation, chromatin profiling, and single-cell or pseudobulk circadian analyses. In this framework, DBP may serve less as a universal therapeutic target and more as a mechanistic indicator of circadian output integrity, helping to explain how circadian misalignment is translated into organ-specific dysfunction.

## Figures and Tables

**Table 1:** Mechanistic determinants of tissue-selective DBP output.

Mechanistic Determinant	How It Shapes DBP Output	Representative Examples and Citations
Rhythmic *Dbp* expression	Determines the temporal window of DBP availability; phase and amplitude may differ across tissues or physiological states	*Dbp* transcription and chromatin transitions at multiple E-boxes [39]; CRY1-dependent phase adjustment of hepatic *Dbp* [74]
Chromatin accessibility and enhancer–promoter architecture	Determines whether a D-box motif or local regulatory element is accessible and transcriptionally competent	Mouse *Dbp* promoter poly(C) motif and hnRNP K support high-amplitude *Dbp* oscillation [70,71]
DBP/E4BP4/NFIL3 balance	Determines whether D-box output is activated, repressed, or temporally reshaped	E4BP4 cycles in near antiphase to DBP/PAR bZIP factors and competes for common sites [41]; functional D-box sequences drive mRNA rhythms [37]
ROR/REV-ERB–RORE context	Modulates upstream clock-gene amplitude and the availability of regulators such as *E4bp4* in a tissue-dependent manner	RORα/RORγ regulate *Cry1*, *Bmal1*, *E4bp4*, *Rev-erbα*, and *Per2* in an isotype- and tissue-selective manner [91]
Tissue-specific signaling and cofactors	Recruits DBP into distinct physiological circuits depending on local signals	TGF-β-linked hepatic retinoid metabolism [85]; ER stress-dependent β-cell DBP/E4BP4 imbalance [95]; estrogen/ERα-linked AVPV *Kiss1* regulation [45]; TGF-β + IL-4-dependent Th9 *Il9* regulation [46]
Target-gene network identity	Determines the functional consequence of DBP activity	Liver *Cyp3a11*/*Cyp26a1*/*Rbp4*-related metabolism [85,96]; β-cell *Arnt* and insulin secretion competence [44,95]; AVPV *Kiss1* [45]; Th9 *Il9* [46]

**Table 2:** Disease and translational relevance of DBP/PAR bZIP-centered output.

Disease/Context	DBP/PAR bZIP-Related Mechanism	Evidence Level	Interpretation
Chronic kidney disease/retinoid metabolism	Hepatic DBP reduction suppresses *Cyp3a11*/*Cyp26a1* and contributes to retinol accumulation	Mouse disease model	Strong mechanistic animal evidence; human relevance requires validation
β-cell dysfunction/type 2 diabetes	DBP/E4BP4 regulation of *Arnt* and insulin secretion-related pathways; ER stress disrupts DBP/E4BP4 balance	Mouse β-cell and disease models; human islet ARNT evidence	Strong mechanistic β-cell evidence; DBP-specific human validation remains limited
Neurobehavioral vulnerability/inflammation	PAR bZIP loss causes seizures; microglial DBP signaling linked to inflammation-related behavioral abnormalities	Mouse genetic and disease models	Strong animal evidence; not yet a standalone human biomarker
Cancer/tumor immunity	DBP opposes E2F8 to promote Th9 *Il9* transcription and antitumor activity	T-cell differentiation and mouse tumor models	Direct immune-oncology mechanism, but human clinical DBP evidence remains early
Cardiovascular pathology	Circadian misalignment contributes to cardiovascular risk; DBP-specific mechanisms are not yet well defined	Human circadian/cardiovascular literature, indirect	Important translational area, but DBP-specific causality should not be overstated
Human circadian disruption	Mistimed or insufficient sleep disrupts rhythmicity and amplitude of human blood transcriptome	Human transcriptomic studies	Supports output-level circadian disruption; DBP-centered signatures remain to be tested

## Data Availability

No new data were created or analyzed in this study. Data sharing is not applicable to this article.

## Abbreviations

The following abbreviations are used in this manuscript:

ARNT	Aryl hydrocarbon receptor nuclear translocator
AVP	Arginine vasopressin
AVPV	Anteroventral periventricular nucleus
BMAL1	Brain and muscle ARNT-like 1
CCL20	C-C motif chemokine ligand 20
CCR6	C-C chemokine receptor 6
CD4	Cluster of differentiation 4
CD8	Cluster of differentiation 8
CRE	CAMP response element
CRY	Cryptochrome
DBP	D-box binding protein
EEG	Electroencephalogram
E2F8	E2F transcription factor 8
E4BP4	E4 promoter-binding protein 4
ER	Endoplasmic reticulum
ERα	Estrogen receptor alpha
GnRH	Gonadotropin-releasing hormone
HIF-1β	Hypoxia-inducible factor 1 beta
HLF	Hepatic leukemia factor
IL-4	Interleukin 4
IL-9	Interleukin 9
LH	Luteinizing hormone
NFIL3	Nuclear factor interleukin 3 regulated
PAR bZIP	Proline- and acidic amino acid-rich basic leucine zipper
PER	Period
PER2	LUC PER2-luciferase reporter
PPRE	Peroxisome proliferator response element
RBP4	Retinol-binding protein 4
REM	Rapid eye movement
RORE	ROR response element
ROR	Retinoic acid receptor-related orphan receptor
SCN	Suprachiasmatic nucleus
TEF	Thyrotroph embryonic factor
TGF-β	Transforming growth factor beta
Th9	T helper 9
TRAF7	TNF receptor-associated factor 7
TTFL	Transcription-translation feedback loop
